# Supplementary figures and images for: Order among chaos: High throughput MYCroplanters can distinguish interacting drivers of host infection in a highly stochastic system
Source: PLoS Pathog. 2025 Feb 11;21(2):e1012894. doi: 10.1371/journal.ppat.1012894 (PMC11813117; doi:10.1371/journal.ppat.1012894)

Rows in 96-well plate

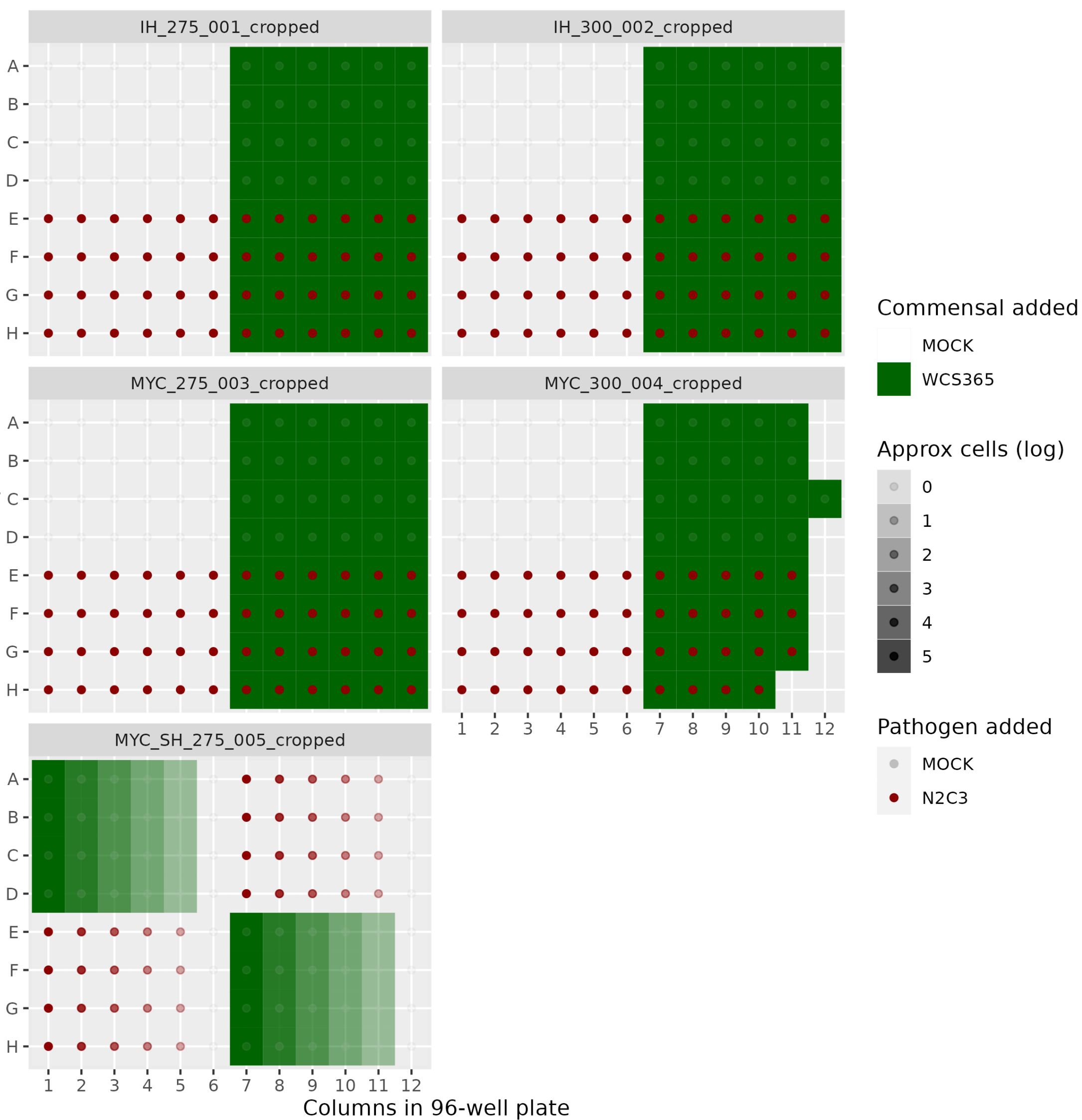

Supplement: S1 Fig — Each panel represents one 96-well plate. Mock treatments were plain 1/2MS 1/2MES pH5.8 plant growth media. (PDF) [file ppat.1012894.s002.pdf]

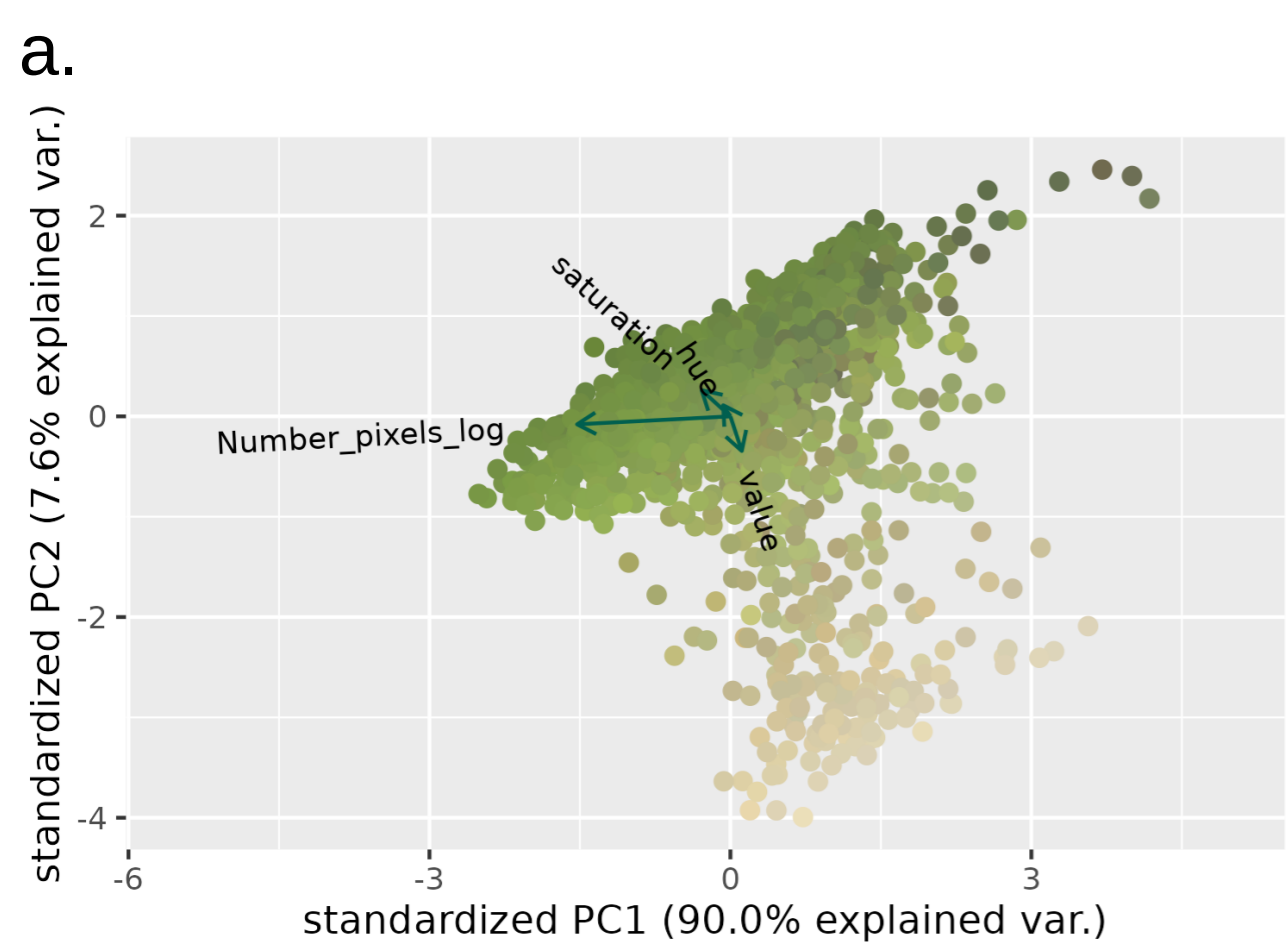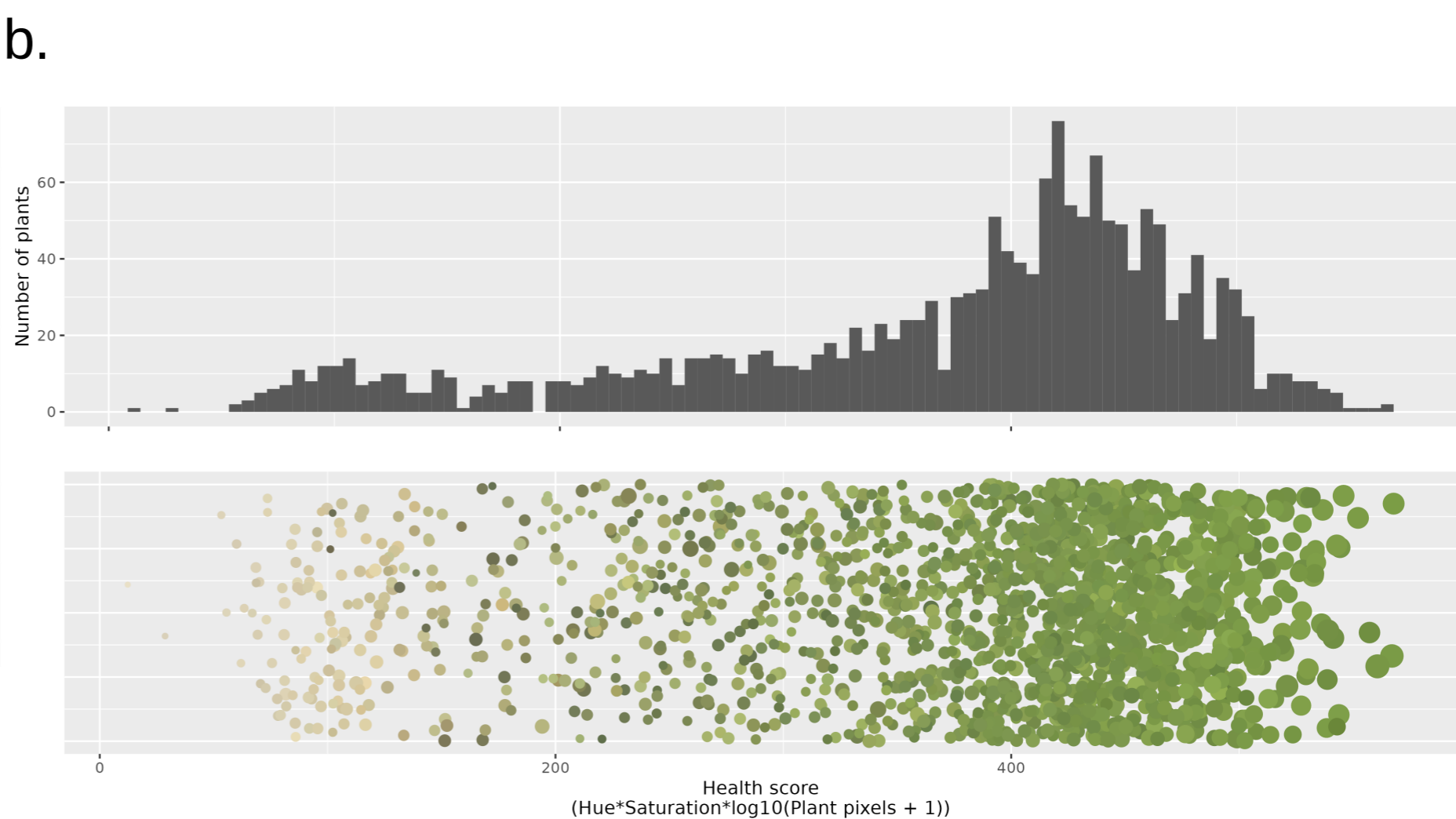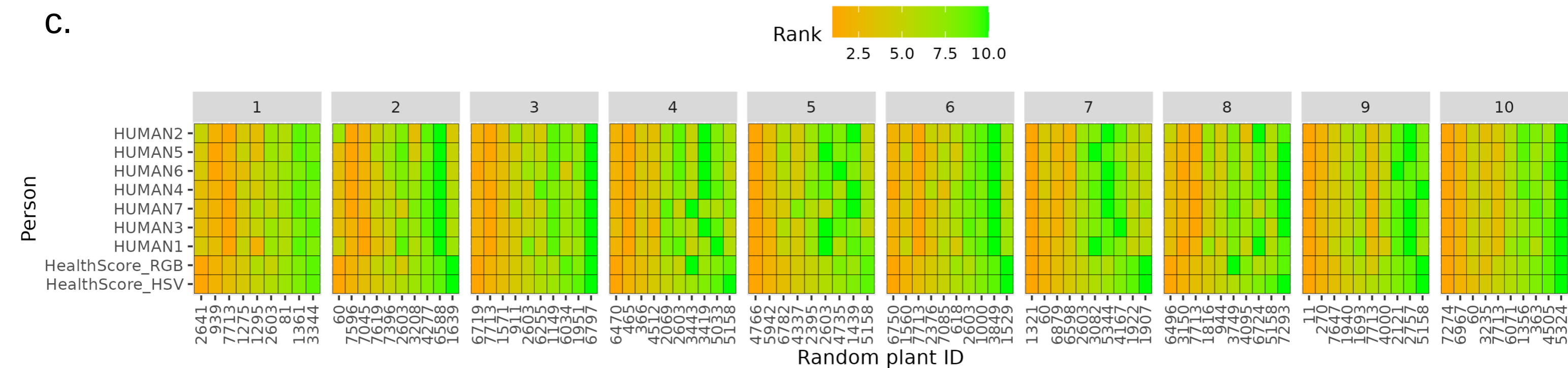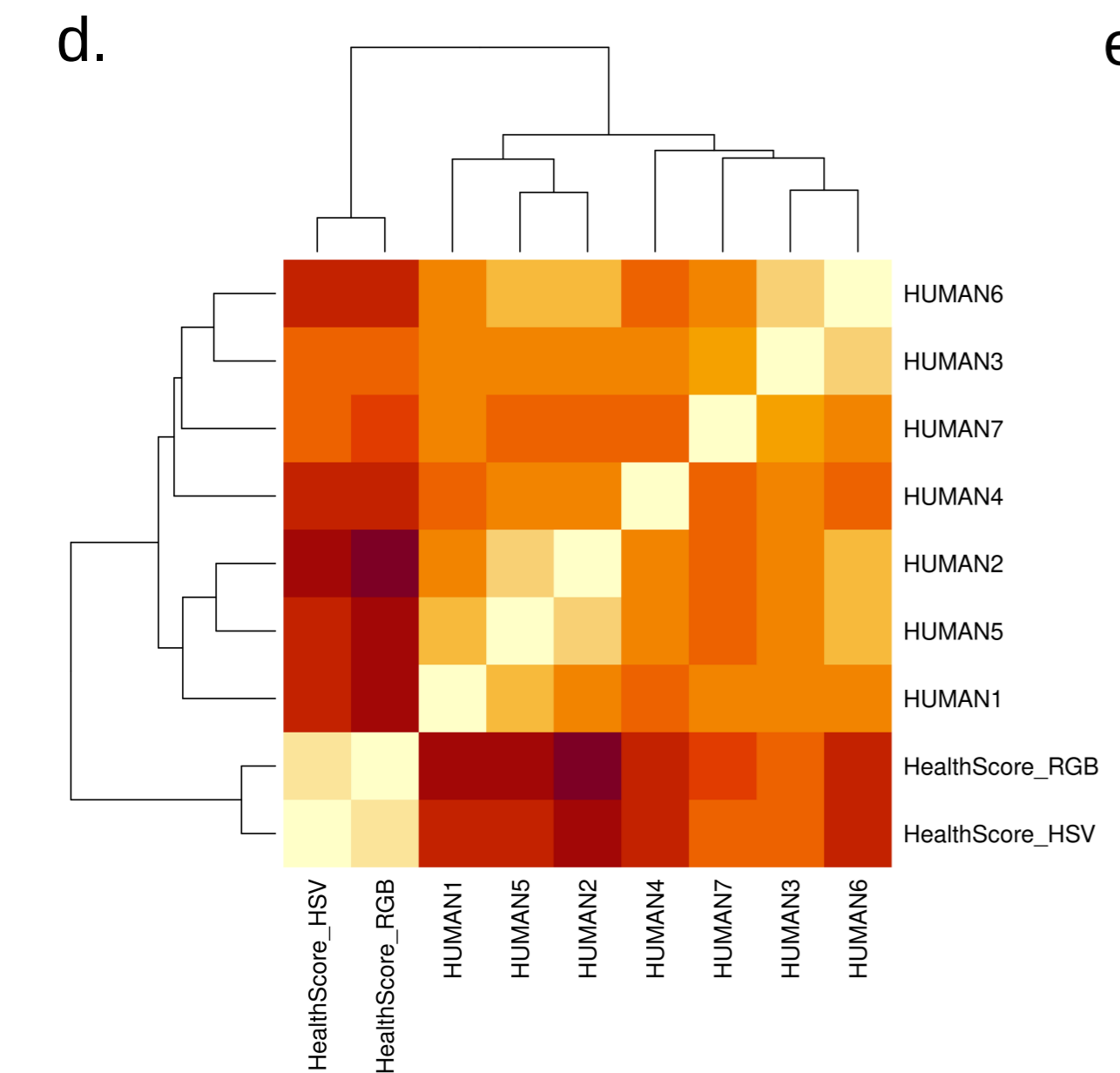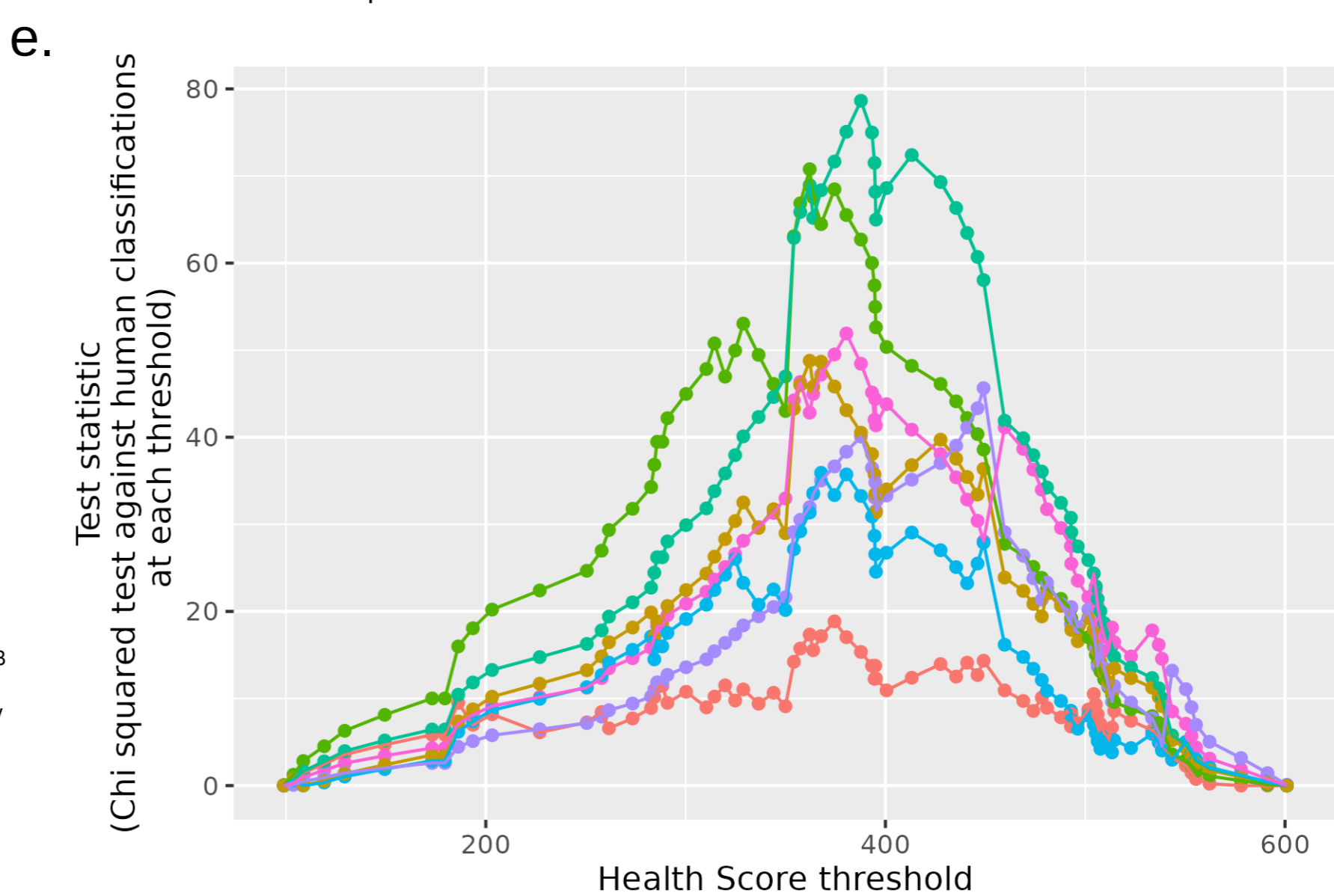

Supplement: S2 Fig — (a) Principal components analysis of median pixel values using the HSV (Hue, Saturation, Value) colour model. Plants are separated by two primary axes: light green/dark green(healthy/stressed) and green/yellow (alive/dead). (b) HSV pixel values were transformed into a single continuous health score metric (hue*saturation*log10(all_plant_pixels+1)*1000), which reflects the roughly tri-modal distribution of plant health states (healthy / stressed / dead). (c) Algorithm-derived “Health Score” metrics using HSV and RGB values were compared to human-derived ranks. Human rankers ranged from lay-people (no scientific background) to expert (PhD). Humans and scoring algorithms were asked to rank 10 sets of 10 plants (85 total plant images; some images were repeated between sets for validation purposes) in order from “least healthy” to “most healthy”. Each was also asked to divide plants into “not healthy” and “healthy” groups. We found that three plants were consistently ranked differently between humans and our algorithm (7714, 3443, 5158), and that these plants were two-toned (half green half yellow). This caused our algorithm results to perform poorly. When these plants were removed, our Health Score algorithm performed comparably to most human rankers. (d) Heatmap of paired Kendall correlations (tau) between each human and/or algorithm plant ranks. (e) “Healthy” / “Not healthy” classifications by humans were compared to algorithm classifications at different Health Score thresholds. To avoid over-interpretation of health score values, we categorized plants as “healthy” or “not healthy” based at a threshold of 400. Binary response variables are used and reported in the main results and text. (PDF) [file ppat.1012894.s003.pdf]

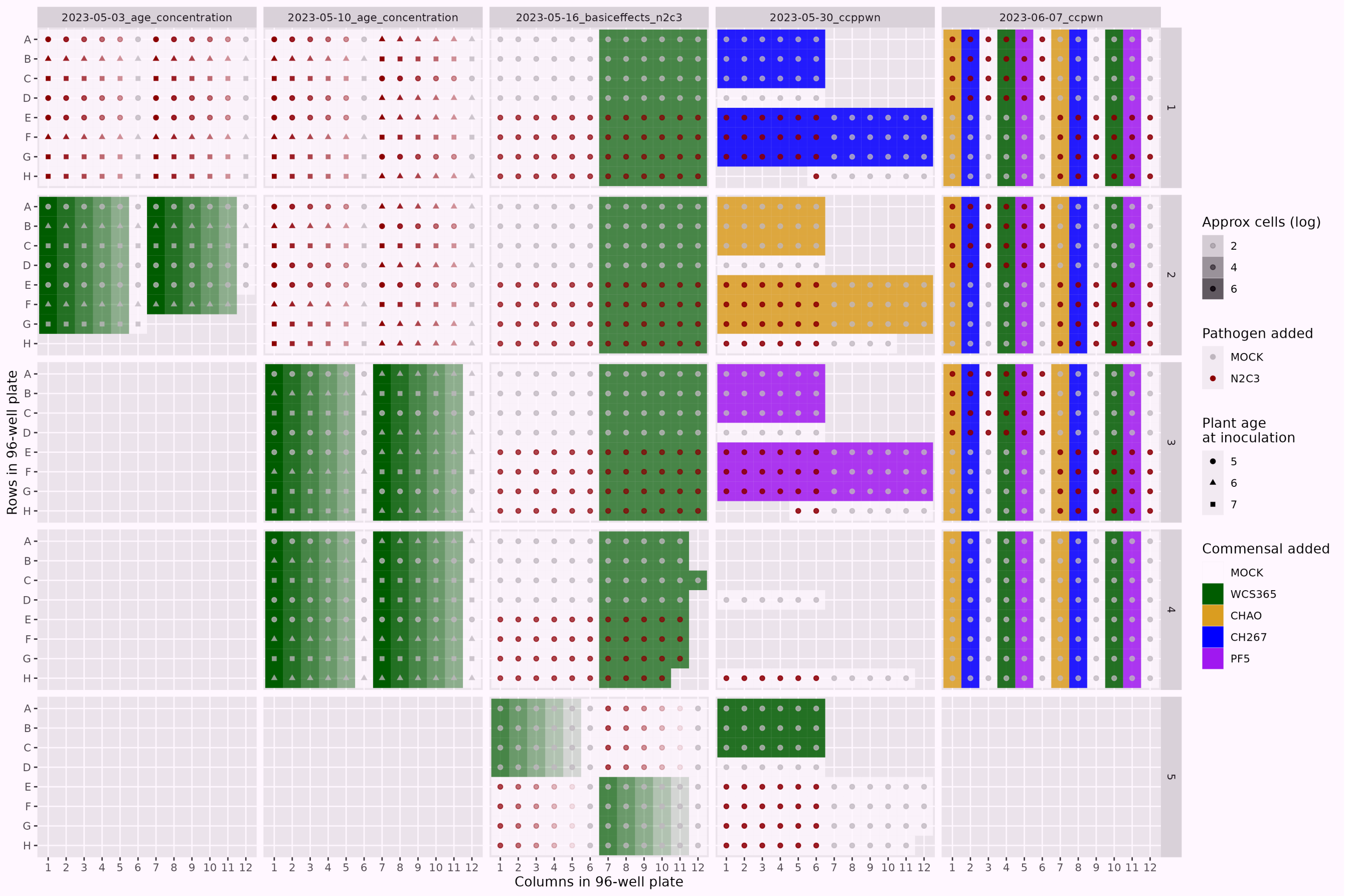

Supplement: S3 Fig — Plants were inoculated at three different ages (5, 6, 7 days) with four different concentrations of pathogen and 4 non-pathogenic bacterial strains across 5 experiments. The breadth of experimental variables used was to increase the range of plant health observed in our pilot data. (PDF) [file ppat.1012894.s004.pdf]

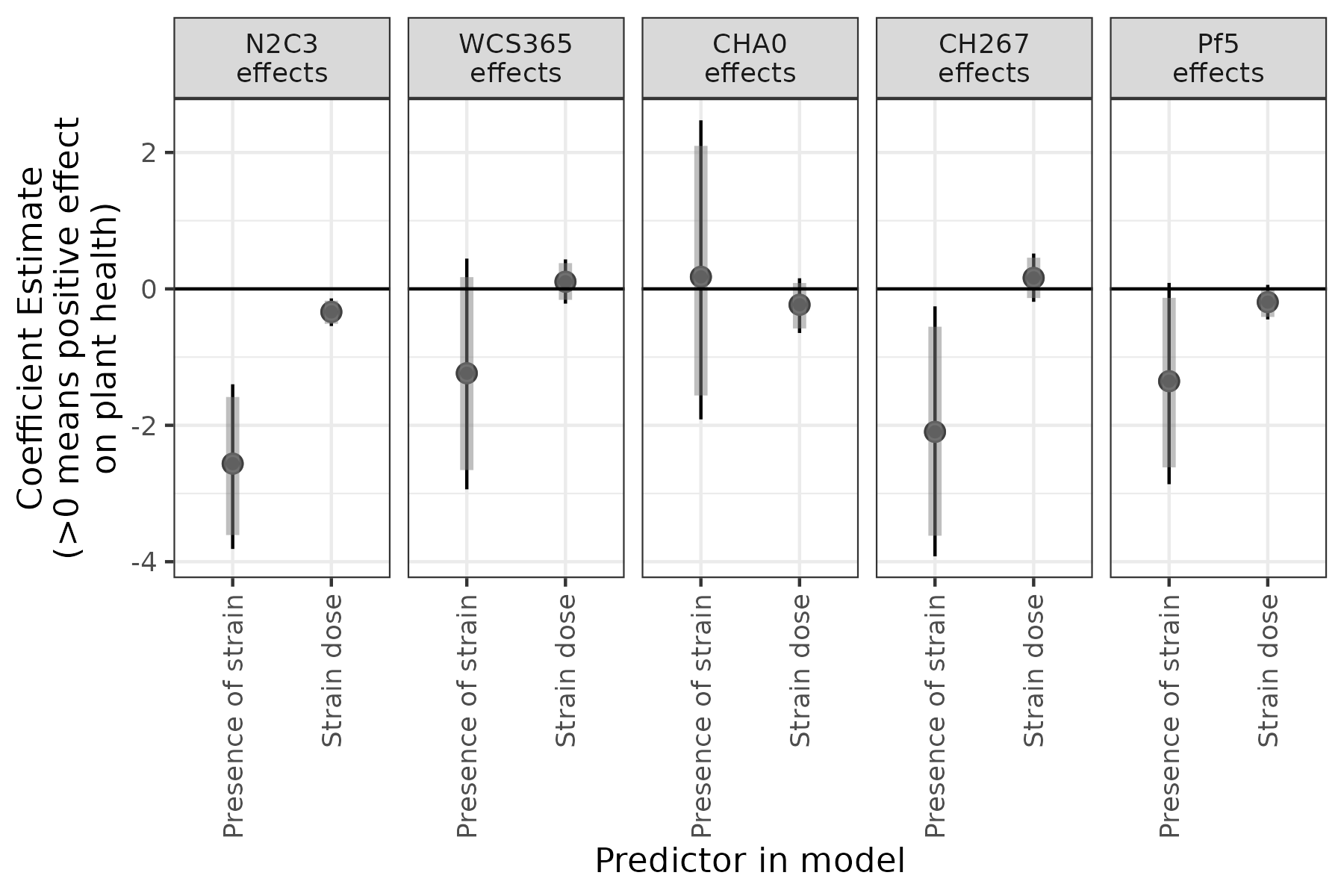

Supplement: S4 Fig — Points are median estimates from a Bayesian Bernoulli model, thick bars are 90% credible intervals, and thin bars are 95% credible intervals. (PNG) [file ppat.1012894.s005.png]

a.

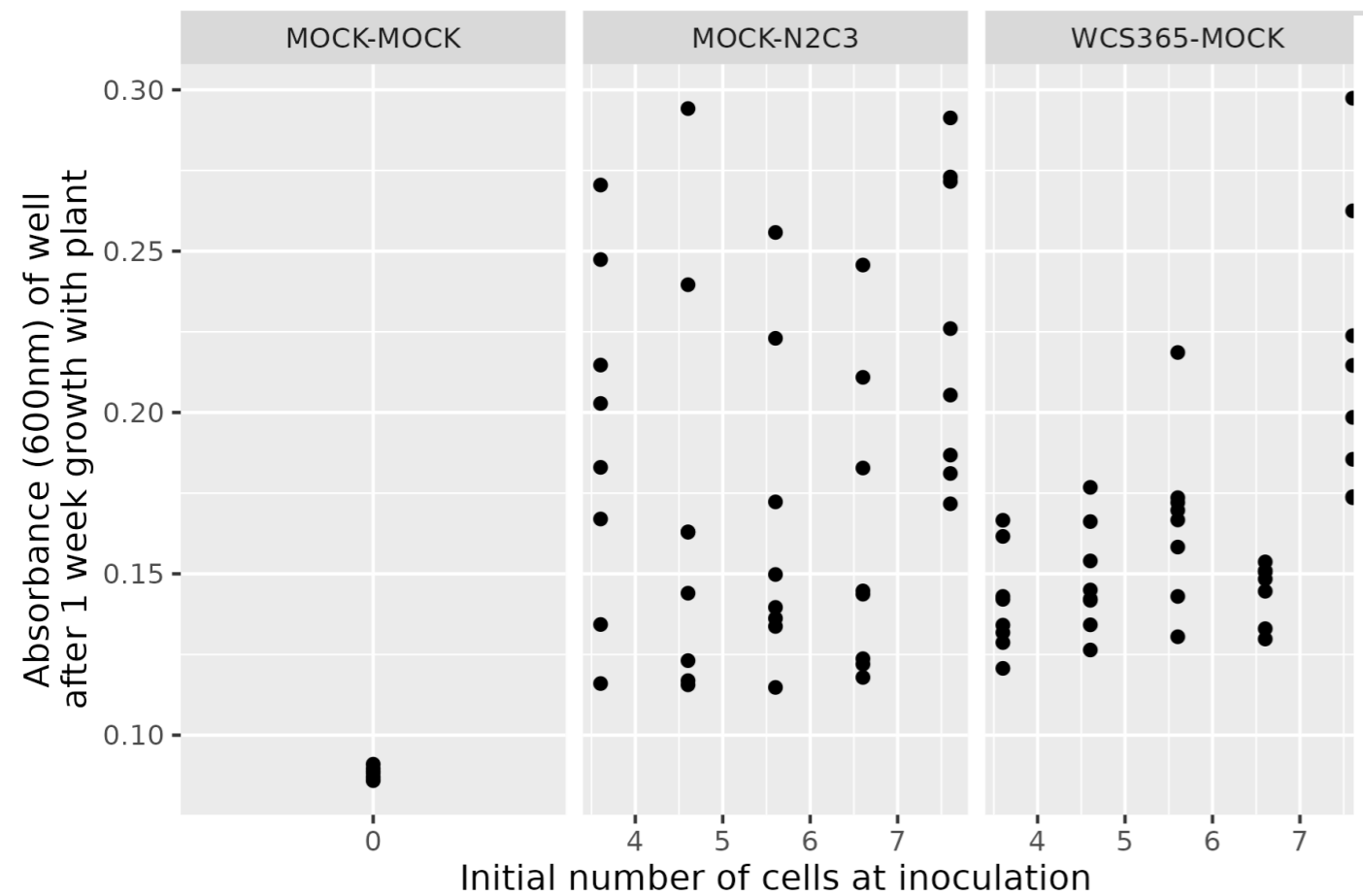

b.

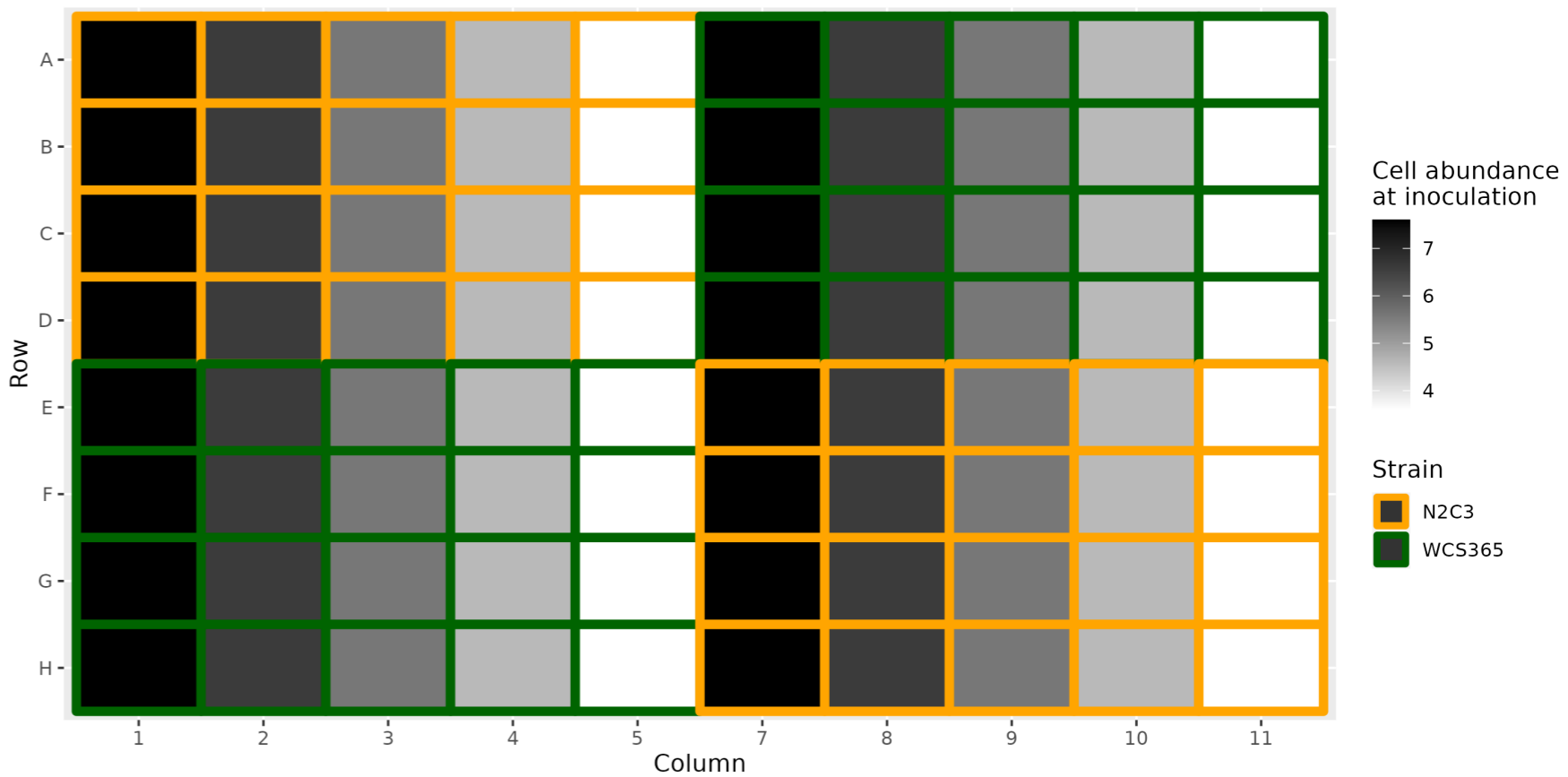

Supplement: S5 Fig — (b) Platemap and experimental design for data shown in (a). Plants were inoculated with different concentrations of pathogen and protective. Cell abundance units are on the log10 scale (1 = 101). (PDF) [file ppat.1012894.s006.pdf]

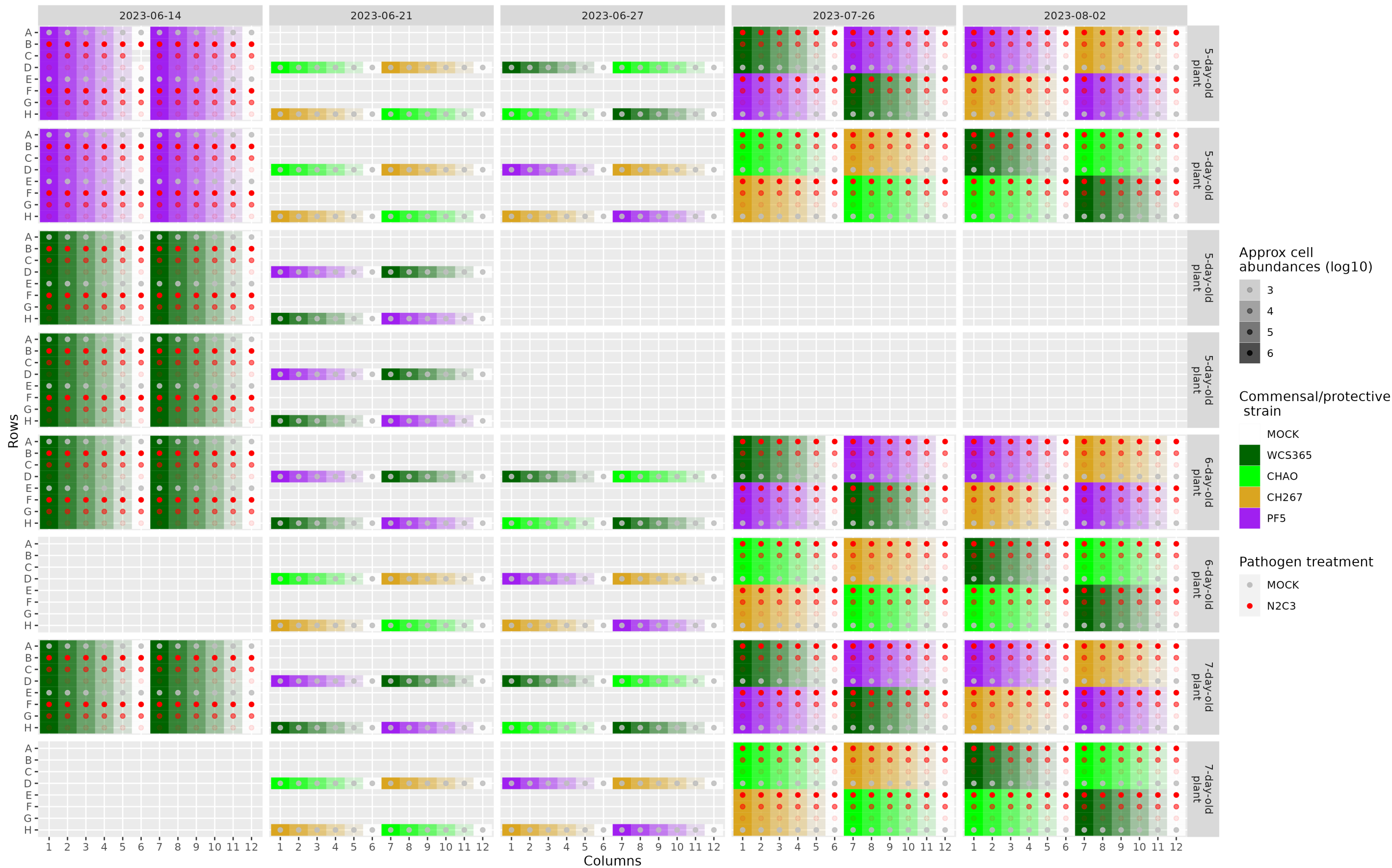

Supplement: S6 Fig — Plants were inoculated at three different ages (5, 6, 7 days) with 4 and 5 concentrations of pathogen and protective, respectively, across 5 experiments. (PDF) [file ppat.1012894.s007.pdf]

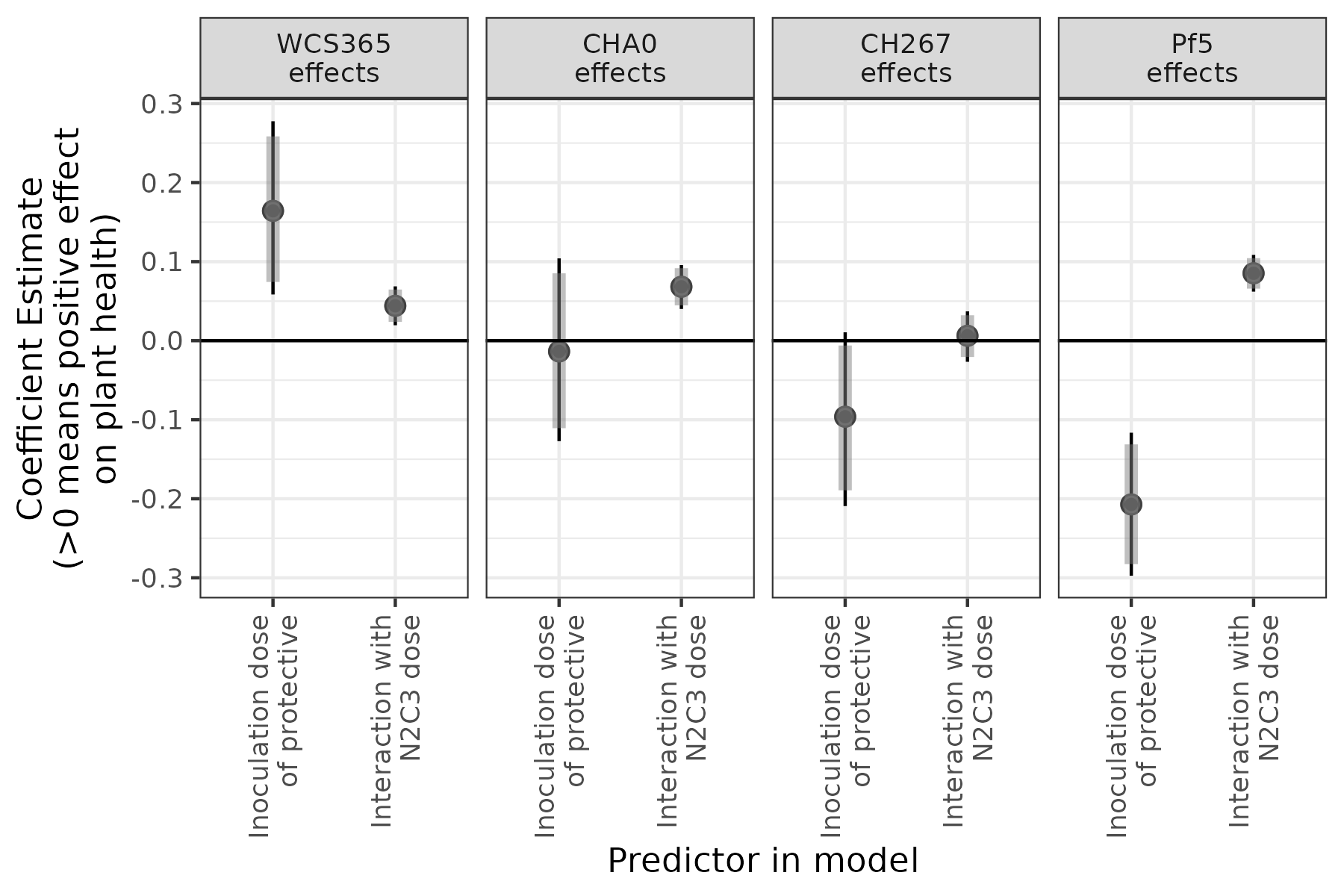

Supplement: S7 Fig — Points are median estimates from a Bayesian Bernoulli model, thick bars are 90% credible intervals, and thin bars are 95% credible intervals. (PNG) [file ppat.1012894.s008.png]

a.

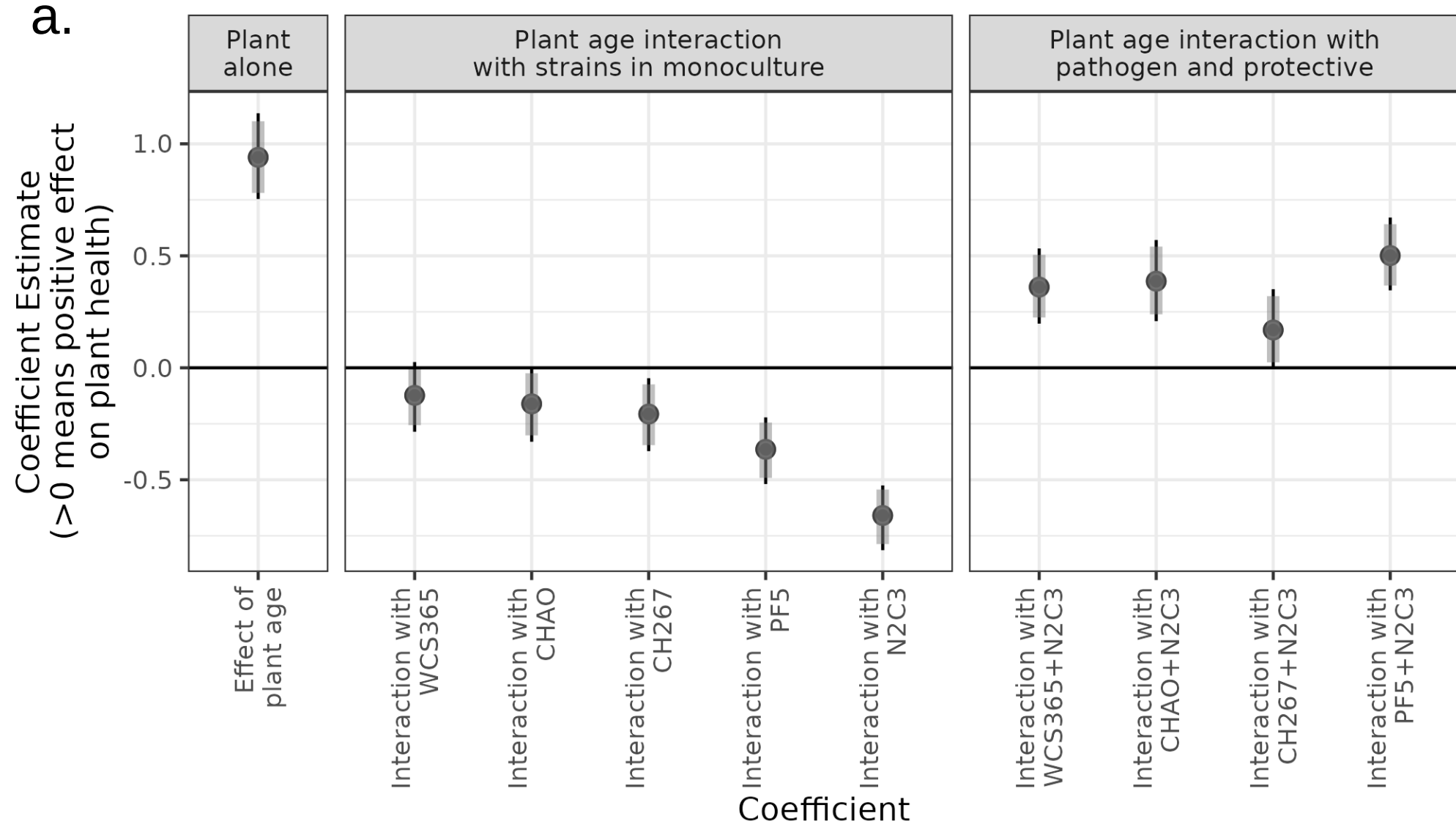

b.

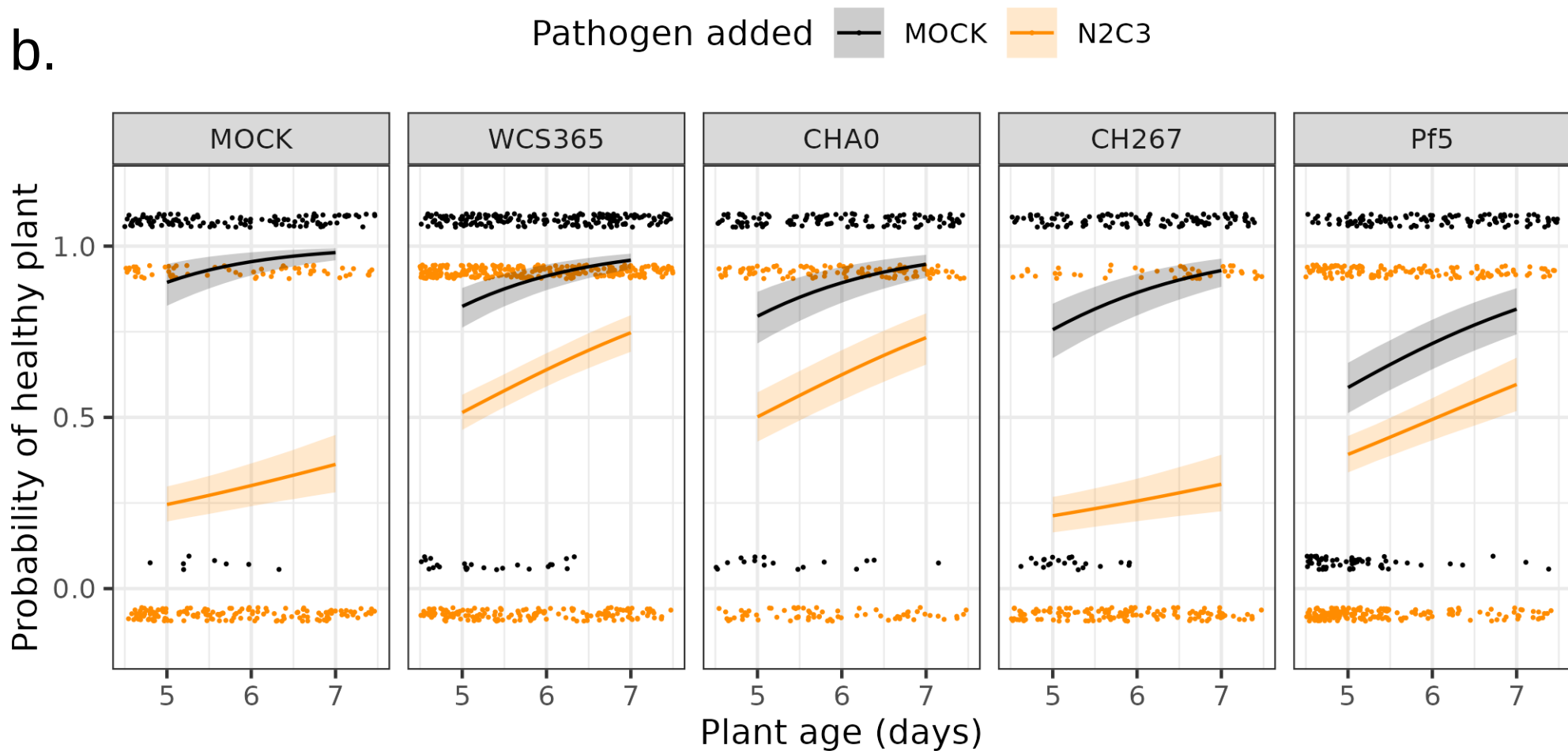

Supplement: S8 Fig — (a) Coefficient estimates for different variables associated with plant age show that the effect of plant age interacts with bacterial strain. In the first panel, the coefficient for the independent (global) effect of plant age is positive, meaning older plants are more likely to remain healthy, overall. In the second panel, we show the interaction between age and each monoculture treatment. Negative coefficient numbers mean there is a weaker effect of age when treated with that strain, relative to the global positive effect of plant age. Here, a negative value for N2C3:plant age means that while plants are usually healthier with age, the effect of plant age when inoculated with N2C3 is less different between 5-day-old and 7-day-old plants (ie 7-day-old plants are not as healthy as one would predict if there was no interaction between N2C3 and plant age). In the third panel, we show the interaction of plant age and each N2C3 + non-pathogenic bacterial strain treatment. When coefficients are positive, it means older plants benefit more from protection of the non-pathogenic strain, relative to what might be expected due to plant age alone. (b) Using the model from (a), we generated prediction intervals for the probability of healthy plants. Dots represent single plants. Lines represent median posterior predictions according to the Bayesian Bernoulli model; ribbons represent 95% prediction intervals. (PDF) [file ppat.1012894.s009.pdf]

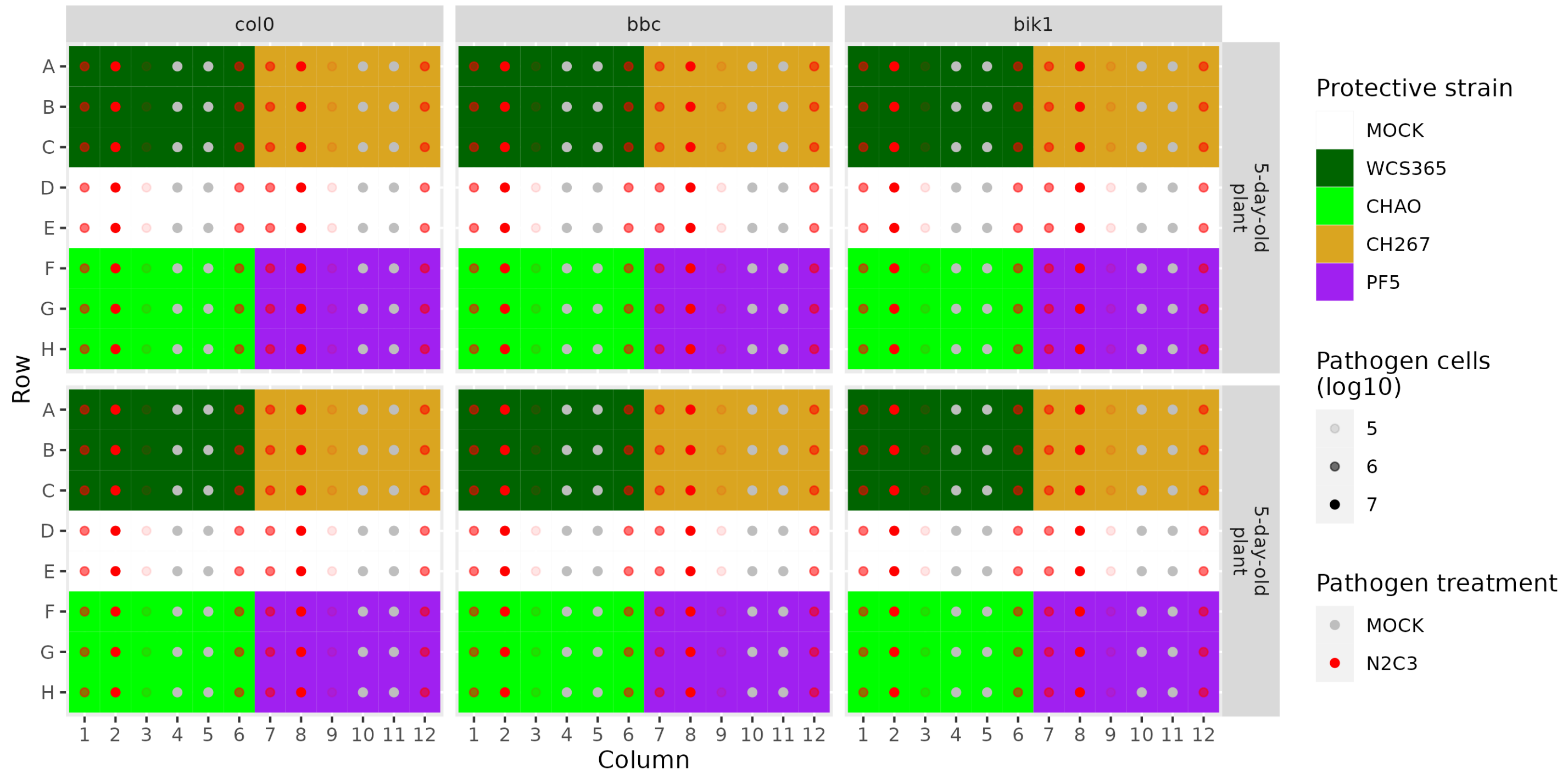

Supplement: S9 Fig — Three plant genotypes (Col-0, bbc, and bik1) were inoculated with WCS365, CHA0, and Pf5 with or without N2C3 at three different protective:pathogen ratios. In our paper, we present only 1:1 and 1–0.1 protective:pathogen treatments because plants from 1:10 treatments were overwhelmingly “not healthy”, which means we could not observe any differences between control (Col-0) and treatment (bbc, bik1) plant genotypes. (PDF) [file ppat.1012894.s010.pdf]

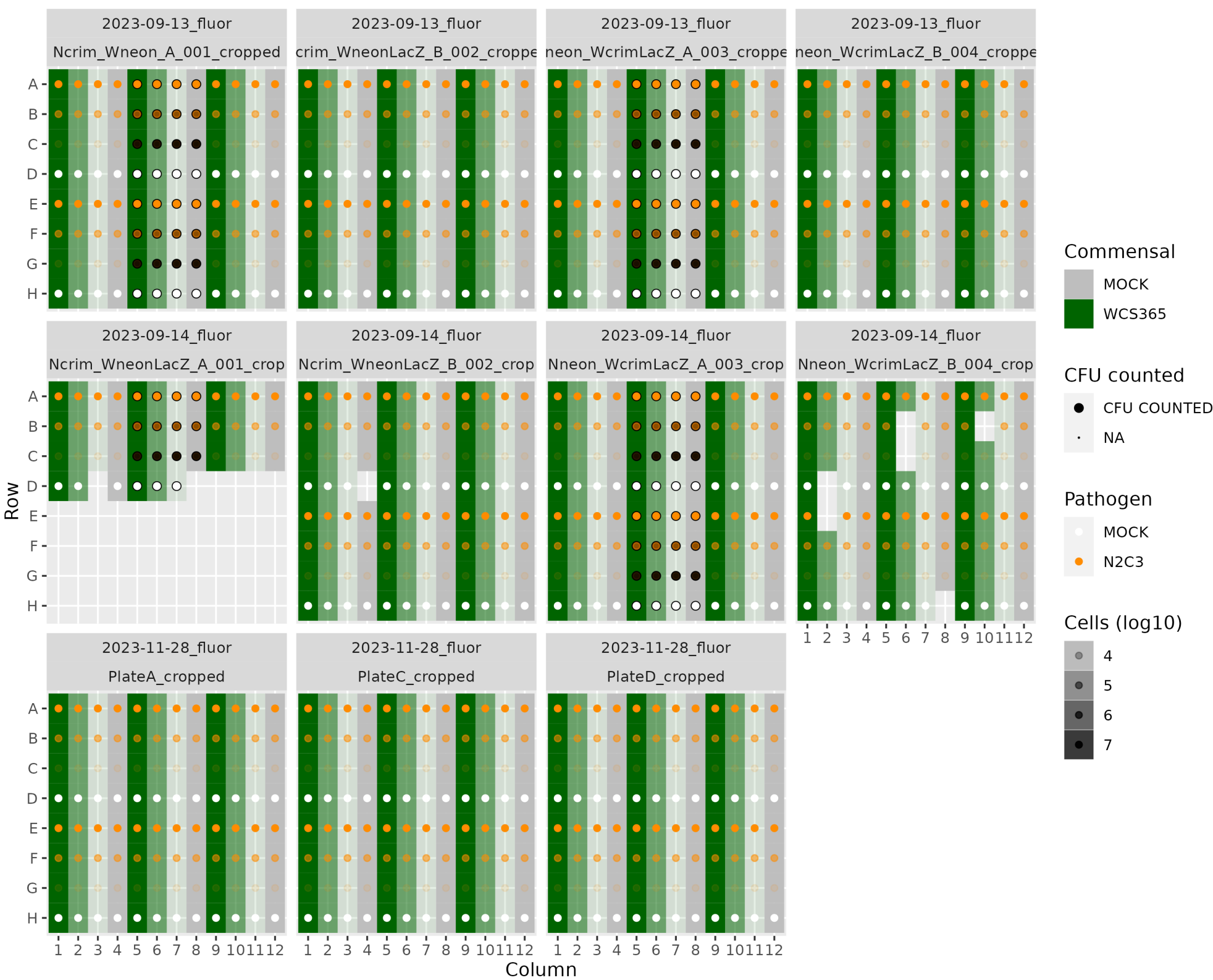

Supplement: S10 Fig — Strains were labelled with either Crimson or Neon plasmids. Black outlined circles indicate wells that were used for serial dilution CFU counting, in order to get lacZ-based estimates of each strain’s cell density, which was fluorescence-independent. Top labels for each panel refers to the temporal experiment, whereas bottom labels for each panel refers to different replicate plates within each experiment. (PDF) [file ppat.1012894.s011.pdf]

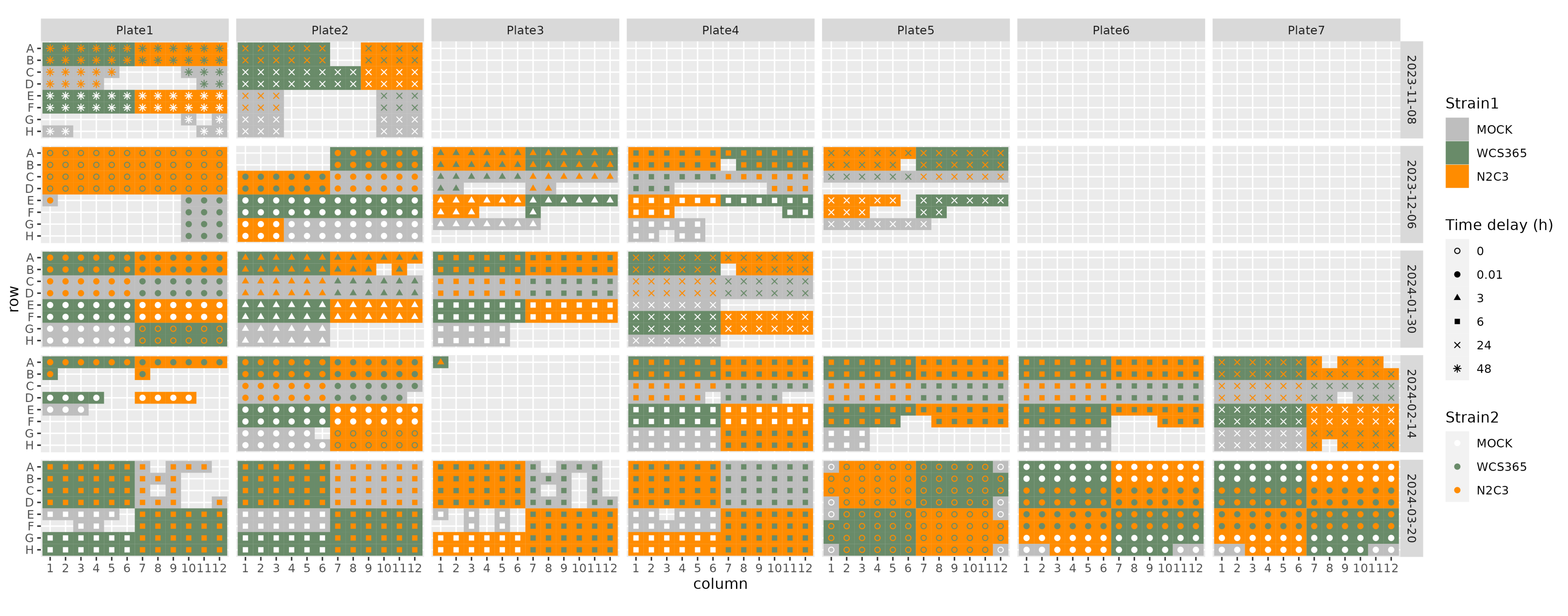

Supplement: S11 Fig — Top labels for panels indicate which plate replicate each panel represents, whereas horizontal rows indicate which temporal experiment each plate belongs to. A time delay of 0.01 refers to “seconds” apart in our dip treatments. A time delay of zero indicates simultaneous inoculation. (PDF) [file ppat.1012894.s012.pdf]

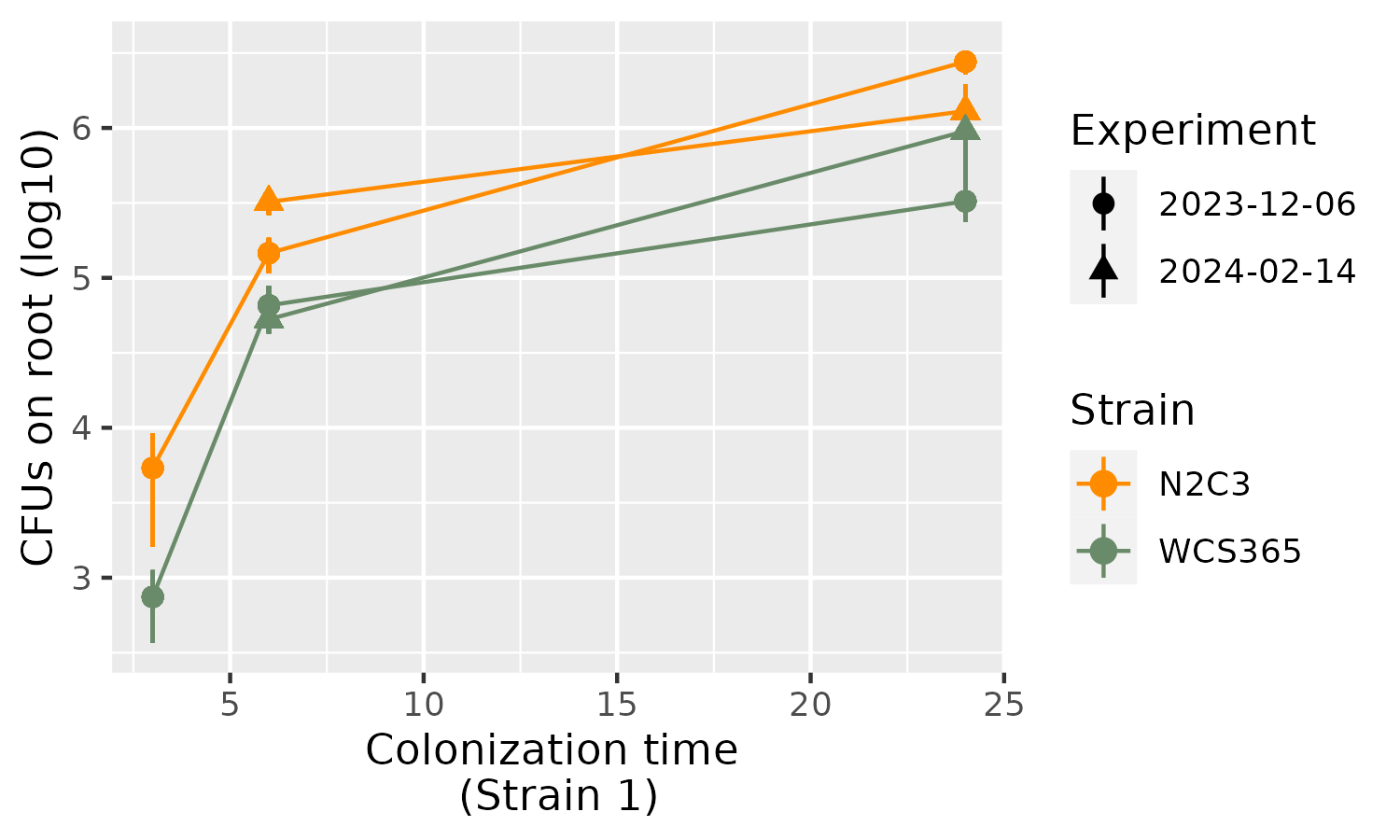

Supplement: S12 Fig — N2C3 reaches slightly higher cell densities than WCS365 on plant roots when in monoculture. (PNG) [file ppat.1012894.s013.png]

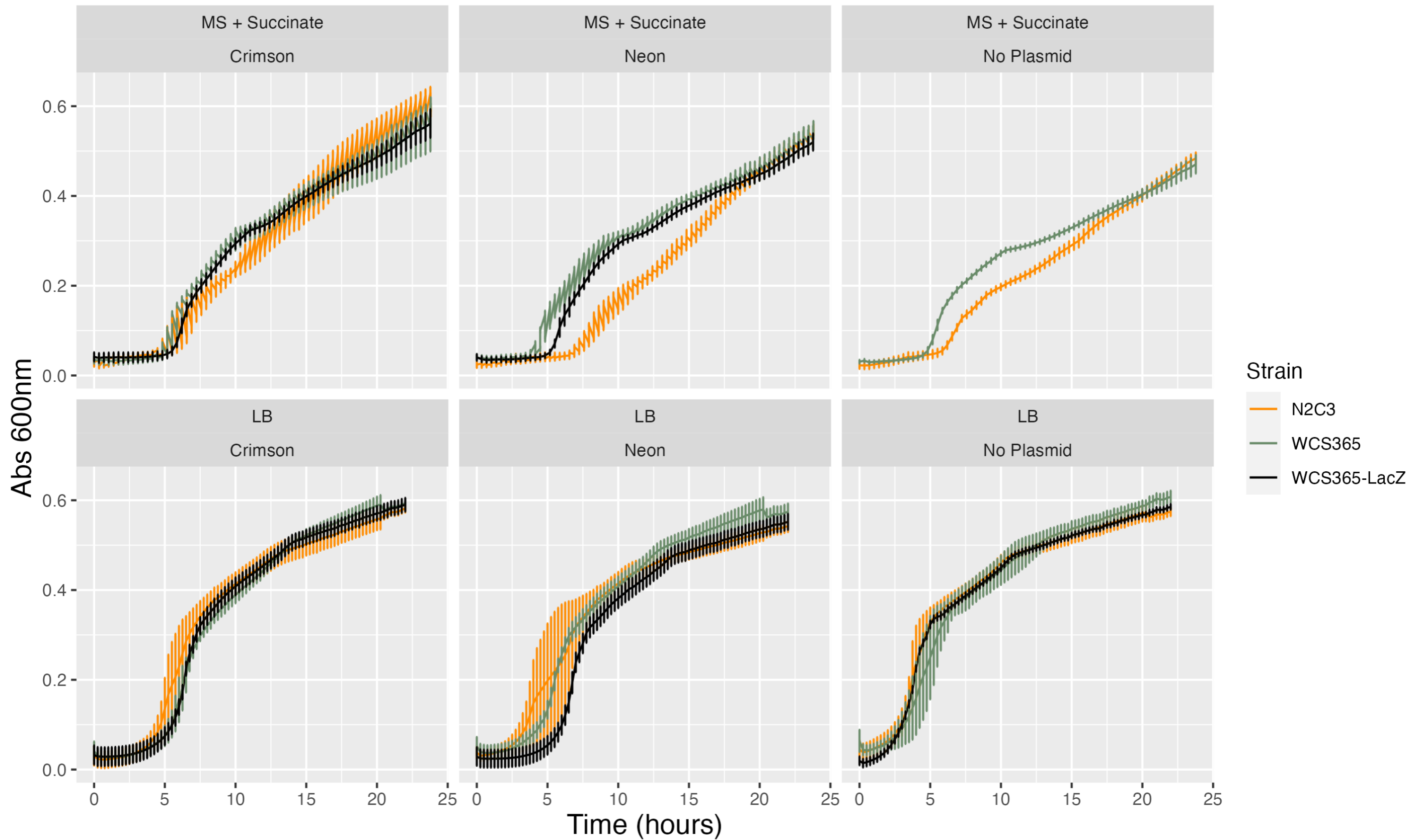

Supplement: S14 Fig — WCS365 and N2C3 do not differ in growth rate or carrying capacity in LB media, with or without plasmids. There is a slight burden on N2C3 with both m-Crimson and m-Neon plasmids in minimal media. (PDF) [file ppat.1012894.s015.pdf]

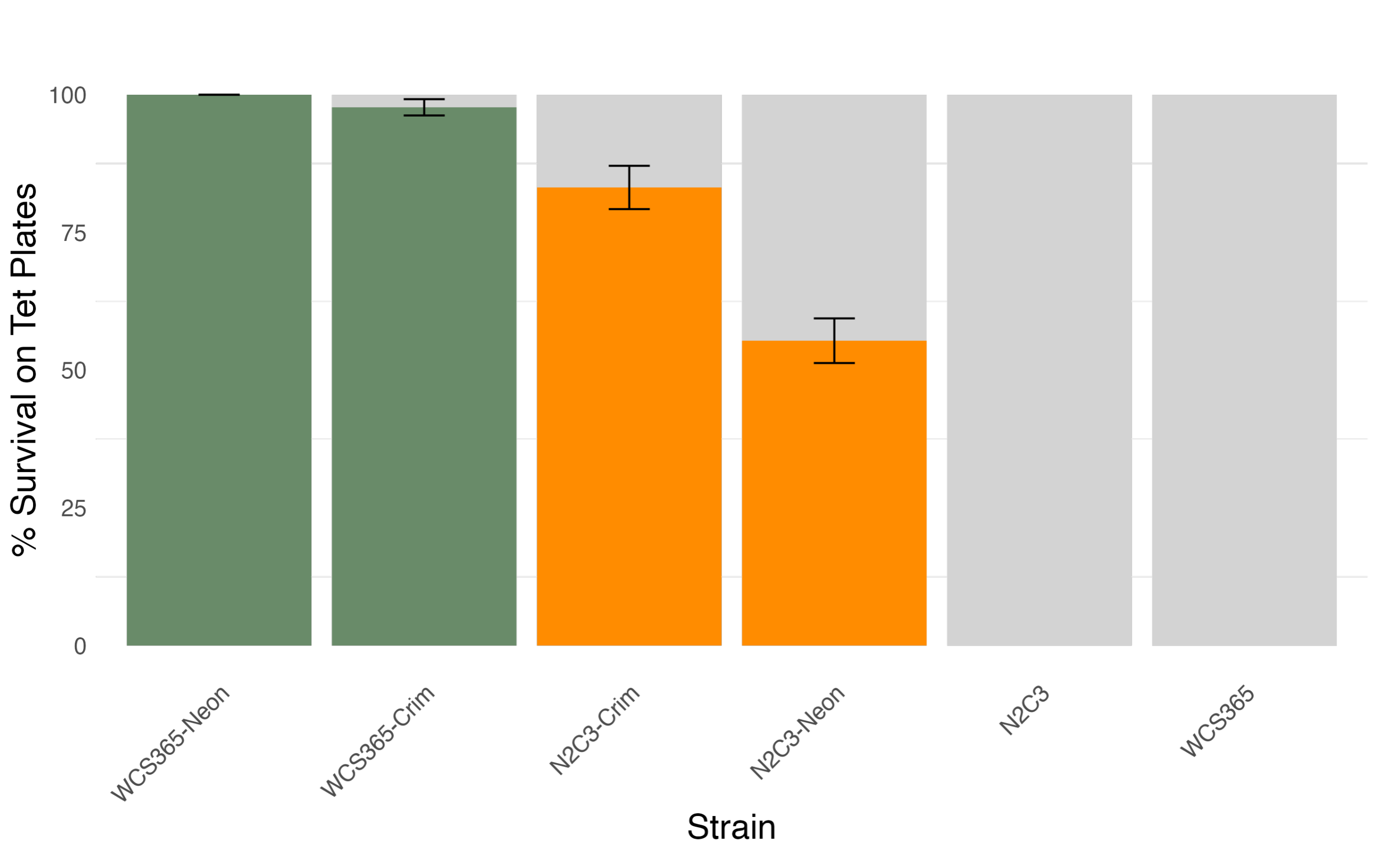

Supplement: S15 Fig — N2C3 strains lost both plasmids at higher rates than WCS365, and this effect was particularly strong for N2C3-Neon. (PDF) [file ppat.1012894.s016.pdf]
