# Supplementary material for: Order among chaos: High throughput MYCroplanters can distinguish interacting drivers of host infection in a highly stochastic system
Source: PLoS Pathog. 2025 Feb 11;21(2):e1012894. doi: 10.1371/journal.ppat.1012894 (PMC11813117; doi:10.1371/journal.ppat.1012894)
Supplement: S13 Fig — Shown are the proportions of WCS365 and N2C3 colonies on solid plant agar when grown with and without plants under different inoculation regimes. Plants were inoculated with WCS365 or N2C3 either at the same time (Concurrent inoculation) or with a 24 hour delay (Delayed inoculation). (PDF) [file ppat.1012894.s014.pdf]

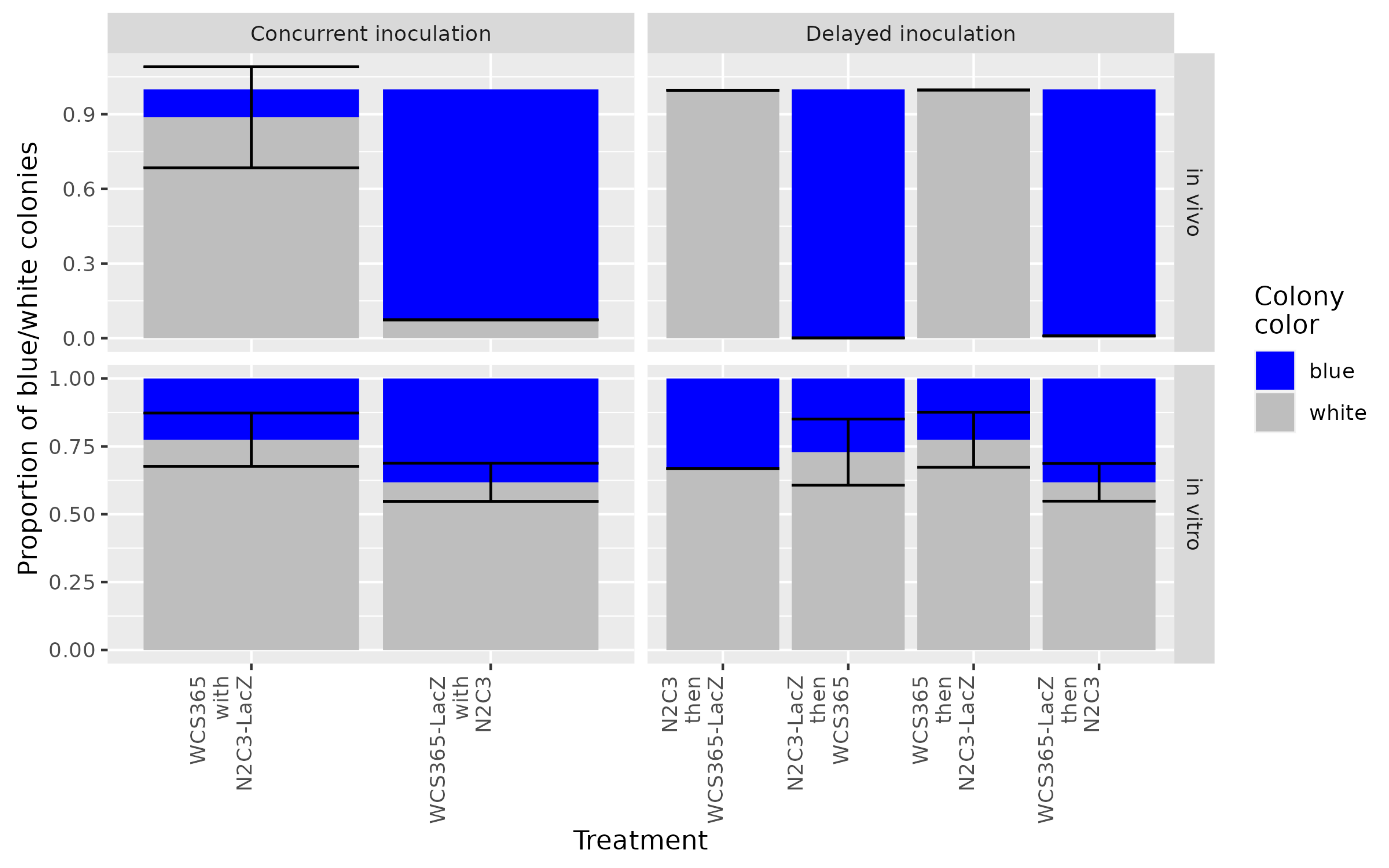
Concurrent inoculation
Delayed inoculation
in vivo
in vitro
Proportion of blue/white colonies
0.0
0.3
0.6
0.9
0.00
0.25
0.50
0.75
1.00
Colony color
blue
white
WCS365 with N2C3-LacZ
WCS365-LacZ with N2C3
N2C3 then WCS365-LacZ
N2C3-LacZ then WCS365
WCS365 then N2C3-LacZ
WCS365-LacZ then N2C3
Treatment
