# Supplementary material for: Order among chaos: High throughput MYCroplanters can distinguish interacting drivers of host infection in a highly stochastic system
Source: PLoS Pathog. 2025 Feb 11;21(2):e1012894. doi: 10.1371/journal.ppat.1012894 (PMC11813117; doi:10.1371/journal.ppat.1012894)
Supplement: Attachment — Submitted filename: pointbypoint_accountofchanges.pdf [file ppat.1012894.s017.pdf]

Point-by-point manuscript account of changes

As this manuscript has been transferred from PLOS Biology, we have made the following changes to the manuscript to fit PLOS Pathogen's initial submission requirements:

> The author "Leah MG Fulton" would like to be changed to "Leah M Fulton" instead.

L35: We have added an "Author Summary", as required by PLOS Pathogens
