# Supplementary material for: Order among chaos: High throughput MYCroplanters can distinguish interacting drivers of host infection in a highly stochastic system
Source: PLoS Pathog. 2025 Feb 11;21(2):e1012894. doi: 10.1371/journal.ppat.1012894 (PMC11813117; doi:10.1371/journal.ppat.1012894)
Supplement: Attachment — Submitted filename: Responses to Reviewers_final.pdf [file ppat.1012894.s018.pdf]

Additional Editor Comments:

Apologies for the delay. Four experts agreed to review this manuscript. However, two reviewers could not submit their reviews timely, but did cause some delay in the decision making process. The two experts who reviewed the manuscript are very positive about this manuscript. Since no experiments are required, the manuscript seems to warrant a recommendation of minor revision. However, reviewer 2 had raised some very critical points that must be addressed by the authors related to microbiome intention with a 2-species approach. While the reviewers and the editors are convinced that this device would be very useful for complex microbiome interactions, the current test is the first step towards testing its full potential in microbiome study. So the logic needs to be thoroughly revised to better justify its potential and the current experimental approach.

Response: We thank the editor and reviewers for the positive assessment of our manuscript. We have addressed the editorial and reviewer comments below and have revised the manuscript accordingly.

Reviewers' Comments:

Reviewer's Responses to Questions
Part I - Summary
Please use this section to discuss strengths/weaknesses of study, novelty/significance, general execution and scholarship.

Reviewer #1: In this manuscript, the authors describe a new platform to increase the throughput of plant-microbe interactions. Further, they use previously characterized interactions to demonstrate a proof of concept of the system to generate robust results, as well as provide image processing pipeline to assign a binary plant health outcome. The high throughput results generated allowed the authors to quantify differences between protective strains against N2C3 infection to investigate a number of variables such as inoculation ratio, timing on inoculation and impact of plant genotype on plant health outcome, despite the biological diversity of plant interactions with multiple microbes. The authors demonstrate that such technical advancements hold the potential to reveal important details in the mechanisms of protection conferred by previously characterized commensal strains.

Response: We thank the reviewer for the positive summary and constructive review of our manuscript. We have addressed your suggested revisions below and made corresponding edits to the manuscript.

Reviewer #2: “Order among chaos: high throughput MYCroplanters can distinguish interacting drivers of host infection in a highly stochastic

system," by Chen et al. describes the novel tool MYCroplanters to conduct simultaneous and sequential strain inoculation experiments in Arabidopsis thaliana. Because their high-throughput tool facilitates experiments, the authors are able to present a significant amount of data demonstrating plant health outcome effects and final occurrence ratio of two inoculated strains, one of which is always the same pathogen strain, and the other of which is one of a variable set of closely related strains. They find that the identity of the paired strain, the ratio at which the paired and pathogen strain are inoculated, and the genotype of the plant alter health outcomes. They also find that the order and manner of arrival of the strains alters final 2-strain relative abundance outcomes, and that plant health outcomes and these 2-strain relative abundance outcomes are linked. In sum, the authors have presented a useful new tool to the community, that also is potentially scale-able or transferrable to other host organisms. I very much enjoyed this paper. I think the method is novel and exciting, and the results are useful in their own right.

Response: We thank the reviewer for their positive assessment of the manuscript and are glad you enjoyed reading it!

My critiques are almost exclusively about framing. I think the framing needs to move away from "microbiomes" and community properties as these are only 2-strain communities, and towards epidemiology – as this is a pathogens journal.

Response: Thank you for this feedback, we have edited the manuscript according to your suggestions below and include a point-by-point response.

Part II – Major Issues: Key Experiments Required for Acceptance
Please use this section to detail the key new experiments or modifications of existing experiments that should be absolutely required to validate study conclusions.

Generally, there should be no more than 3 such required experiments or major modifications for a "Major Revision" recommendation. If more than 3 experiments are necessary to validate the study conclusions, then you are encouraged to recommend "Reject".

Reviewer #1: Because there is such an impressive number of variables tested, the text could provide a bit more clarity to help the reader follow all what the discussion of each presented experiment:

1) It might be easier to follow the text describing the different microbiota and plant genotype variables if "strains" was used to describe the different

Pseudomonas strains and "genotypes" was just used to describe the Arabidopsis genotypes.

Response: Thanks for this suggestion, we have edited the manuscript to refer to bacteria as strains instead of genotypes.

2) In Figure 3, the orange and yellow data under the "No microbiota" heading are a bit confusing because the ratio of 1:1 and 1:0.01 are really 0:1 and 0:0.01. Perhaps this would be more clear if there was a different key and/or color code from just the "No microbiota" graphs.
Response: Thanks for this suggestion, we have changed the colors on the no non-pathogenic graph and added a corresponding key and legend.

3) Figure 5b have pretty extensive label names, a little schematic added to the top or bottom of this graph might make these different conditions more intuitive.
Response: Thanks for the suggestion; we have added a schematic to make this clearer and shortened the axis labels.

Reviewer #2: First, the abstract and 2nd paragraph of the introduction led me to expect that this manuscript was about manipulating lots of diverse microbiome communities, yet, that is not what is happening. Referring to these 2-strain mixes as “microbiomes” is confusing -- I also hesitate to call them “communities.” Using “protective strains” or “commensal strains” or “non-pathogen strains” or even “microbes” or similar instead of “microbiota” and “microbiome” in the abstract and elsewhere would be more transparent. Similarly, “community structure” and “community dominance” don’t make much sense in a 2-species context, as there is simply relative dominance of one strain versus the other. Again similarly, the fact that final relative abundance of a pathogen and non-pathogen in a 2-strain community and host health are correlated does not seem particularly surprising. Framing the 2-strains as a “community” with “composition” that predicts “infection outcome” seems to inflate the importance too much. However, I *am* completely convinced that this device would be very useful for complex microbiome interactions, and I appreciate the text pointing that out.
Response: Thank you for this comment and suggestion. We have edited the text to refocus on virulence and disease, and the role of single strains of microbes in protecting against disease. We opted to change the term for microbiota to “non-pathogenic” instead of “commensal” or “beneficial” strains because “non-pathogenic” encompasses microbes of diverse lifestyles. We have clarified throughout that we mean individual strains, or 2-member communities.

Second, and this is key given the journal, I think the authors should add motivation and interpretation of this study in light of epidemiology and

disease (theory/modelling or etc.). Intra-host competition strains seems very related to the design and results, maybe that would be a good framework.

Response: Thank you for this suggestion, we edited the manuscript (abstract, intro and discussion) to reframe the manuscript to focus on disease and epidemiology.

Third, given that MYCroplanters are one of the key items presented in the manuscript, I think it is important to provide more discussion about these and their utility. I'm glad to already see a blueprint-style figure & the 3-D printing files. In the supplemental figures, it looks like most treatments had to be applied in spatially grouped ways -- presumably because it is difficult to prevent microbial cross-contamination between wells. I think a (brief!) discussion of the trade-offs in experiment size and the ability to randomize, or the statistical tools used to deal with the blocking/plate/edge effects that might be necessary is warranted.

Response: Thanks, we have have added a few sentences to the methods and discussions. First, it is not difficult to prevent cross-contamination between wells; strains were often grouped for ease of inoculation with multichannel pipettes favoring reducing human error over randomization. To mitigate edge effects, we inverted rows (A-B-C-D-E-F-G-H, E-F-G-H-A-B-C-D) and columns (1-2-3-4-5-6-7-8-9-10-11-12; 7-8-9-10-11-12-1-2-3-4-5-6) between replicates within the same temporal batch. To reduce plate effects, we switched which strains were on the same plate between temporal batches where applicable (e.g. WCS365 and CHA0 share a plate in experiment 1; WCS365 and Pf5 share a plate in experiment 2). For future work, we note that the setup is compatible with liquid handling robots, which makes full randomization possible.

Part III – Minor Issues: Editorial and Data Presentation Modifications
Please use this section for editorial suggestions as well as relatively minor modifications of existing data that would enhance clarity.

Reviewer #1: There are also a couple of labeling suggestions:
1) Figure 3 x-axis labels should be italicized for the genotypes, with the WT being labeled "Col-0" as it is in the text.
Response: Corrected

2) In Figure 5a, the times besides the dips have units. The authors provide details on what "dip" means in the text, but perhaps for consistency the "dips" could be relabeled times with unit, such as "3 seconds" or "< 3 seconds".
Response: Thanks, we have added units for clarification.

Reviewer #2: The side panels on figure 4 are a clever and intuitive way to deal with lack of detection. However, why is the x-axis arranged as it is? It would make more sense in numerical order
(100000-1, 100-1, 1-1, 1-100, 1-00000) especially because the results are interpreted that way.
Response: Thanks, we have reordered the data on the figure.

The figures all use the “probability of plant health” or similar, but the manuscript also uses the clearly related “health outcome” as well as less clearly related “infection rate” and “infection outcome”. I think reducing the number of different phrases would be helpful.
Response: We have removed “infection outcome” and reframed the majority of mentions to be “probability of plant health” or “plant health”.

Sometimes “dose” is refers to total amount, and sometimes to ratio. Since the results depend on the type, it might be best to use always “total dose” or “dose ratio”. E.g. L303 I think the significant result was for ratio not total dose?
Response: Thank you for catching this, we have corrected instances when we meant ratio.

Lines 109-110 in the abstract. I’m uncomfortable with community structure framed as a predictor in the same way as the manipulated variables. The 2-species composition outcomes were also observed, and therefore, we do not know the nature of the relationships between the final composition and plant health. Granted, we have a strong prior here, but it could also be the case that declining plant health allows spikes in pathogen numbers.
Response: Thank you, we have reframed the manuscript in light of plant health and disease and decreased mentioned of microbiome and community structure (or clarified when we mean a 2-member community).

Line 153: Commensal is usually reserved for organisms with truly no effect on the host. It seems these strains may be better described as conditionally mutualistic?
Response: Thanks, we have opted to use “non-pathogenic” instead of commensal or mutualist since indeed the lifestyle changes depending on the condition.

Lines 192-196: These summarize content already introduced and could be skipped.
Removed

Line 243: I suggest elaborating on “distinct engineering strategies” or skipping
Removed

Line 248: I could not easily figure out which of the experiments these data came from.
Response: The are from Figure S8; we have added an additional reference to the supplementary figure.

Line 408: To me "confounded" communicates that priority effects are artificially inflated. Comparing a with b, it looks like the dip + move to the second well with many microbes "eroded" priority effects.
Response: We intended to say that finding that priority effects are occurring, might be muddled or invalidated by the observation that there are also increased cell numbers over time (i.e. a change in ratio). To clarify, we removed this from the figure 5 legend; we have a more extensive explanation of our meaning in the results section starting on line 460 where we outline two competing hypotheses.

I don't understand the separation of priority and inoculation effects. I do think the analytical separation makes sense, and that there is a neat result here, but I'm not sure the words to describe it in lines 419-433 make sense. Isn't getting early access to host nutrients and therefore increasing in population part of what a priority effect is? Also "physcially precluding" the first strain seems to fit more with the "neutral assembly" process because physically precluding would still be just a numbers game? Maybe the early microbes alter how the plant develops & therefore fundamentally alter the habitat (i.e. like ecosystem engineering??). I think I'm missing something the authors could easily explain better.
Response: Thanks for highlighting this; we have reworded this section to hopefully improve clarity. In short, we are trying to differentiate between two possibilities: strain competition outcomes are a result of neutral assembly (by definition, no competitive differences between strains, and therefore success is proportional to relative abundance) OR outcomes are a result of priority effects (which may have many mechanisms, one of which is physical preclusion and nutrient pre-emption). Under this framework, physically precluding a second strain from invading does not represent neutral assembly because it is by definition not neutral-- the first strain has a competitive advantage.

Thus, in order to test whether priority effects are truly occurring (and it is not just a result of different population sizes at inoculation), we vary the second strain dose to capture the relationship between population ratio and strain dominance-- and determine whether their relationship aligns more closely with priority effects (horizontal line) or neutral assembly (diagonal line).

Lines 494-503: in 7b, the relationships all overlap, which fits with this conclusion. However, in 7a, the relationship between final ratio and plant infection differ depending on the treatment. It seems then that the

conclusion should be that inoculation ratio shifts (but only slightly) the nature of how strain ratio predicts infection.
Response: Thanks for taking the time to consider this figure so carefully- we believe you might be referencing the shifts in the dotted / dashed / solid grey lines in Figure 7b, which indicate differences between total cell abundance across treatments, and not inoculation ratio. For example, inoculating N2C3 and WCS365 at 0.01Abs and 0.01Abs; versus at 0.001Abs and 0.001Abs, respectively would yield the same inoculation ratio but different total cell abundances. In this sense, you are correct that “treatment” has a slight effect on plant health, in that more total cells tend to make plants sicker. For Figure 7b, we do not explicitly discuss the effect of cell load, but show the subtle effect of cell load for transparency sake. Our intention behind this figure was to demonstrate the robust relationship between plant health and final strain ratio (x-axis). Since inoculation ratio (colours) and final strain ratio (x-axis) are obviously correlated, we use a LOO (leave-one-out) model comparison to show that final ratio is, in fact, the better predictor of the two ratios (shown via statistical results, but not through the figure directly). These statistics are included in the text of our manuscript. We hope this clarifies our discussion surrounding this figure panel.

Line 706: Is this missing the absorbance value?
Response: We aren’t sure what the reviewer is referring to here; there is a mention of fluorescence on this line, and the excitation/emission settings were defined in the previous section (line 703).

L915: probably seeds not seedlings were made axenic with Cl gas?
Response: Yes, thank you, corrected.
